# Novel mesoporous silica nanocarriers containing gold; a rapid diagnostic tool for tuberculosis

**DOI:** 10.1186/s12906-021-03451-7

**Published:** 2021-11-05

**Authors:** Chang Sun, Xiaoying Zhang, Jialu Wang, Yahao Chen, Cunren Meng

**Affiliations:** Xinjiang Medical University First Affiliated Hospital Urumqi, Xinjiang, 830011 China

**Keywords:** Mesoporous silica nanoparticles, Gold nanoparticles, Tuberculosis, Rapid diagnosis, Calorimetric evaluation

## Abstract

Tuberculosis (TB) is major health concern and reason of deaths from decades to current date. Even though with a lot of advancements, diagnostic techniques, and discovery of standard antibiotics TB remains crucial challenge and can create worst scenario for human health in near future. Nanoparticles play emerging role in diagnosis and treatment of TB. In this study, we developed mesoporous silica nanoparticles containing gold (MSNs@GNPs) for rapid diagnosis and treatment of TB. The physicochemical characterization revealed effective surface morphology and particles diameter, that is applicable for in vitro applications. The in vitro antimicrobial analysis revealed that the designed MSNs@GNPs has retained significantly lower minimal inhibitory concentration (MIC) values and can effectively demolish *mycobacterium tuberculosis (Mtb)*. Furthermore, the diagnosis efficiency of the MSNs@GNPs was evaluated by calorimetric analysis. Which demonstrates that MSNs@GNPs can be used for rapid diagnosis of the tuberculosis when applied on in vitro culture of the *Mtb*. The current study needs further verification on human’s clinical samples from tuberculosis patients. However, MSNs@GNPs can be a versatile clinical approach for the rapid diagnosis and clinical treatment of the tuberculosis.

**Supplementary Information:**

The online version contains supplementary material available at 10.1186/s12906-021-03451-7.

## Introduction

Tuberculosis (TB) is a deadly contagious disease occurs due to the bacteria *Mycobacterium tuberculosis (M. tuberculosis)*, affecting lungs (pulmonary TB) but can also affects central nervous system (CNS) causing severe meningitis. TB is a sever type of infection affecting 30 % of the world’s population [1]. The incidence of occurring TB is increasing progressively and demands new diagnostic tools and treatment regimens. While treating TB, antimicrobial resistance is a head tackling problem and leading to severe human’s health risk [2, 3]. The problem of early diagnosis is aggravated by the reality that no rapid diagnosis exists in the clinics up to date, along the absence of new antibiotics since decades. The increasing incidence declare inefficient and rapid diagnostic procedures and lack of satisfactory treatments [4–6]. There is an unmet need of the designing new diagnostic and treatment approaches to overcome the TB related hazards in order for smooth treatment and prevention of TB. Ideally approaches should be made for the development of sensitive, cost-effective and rapid diagnosis, treatment and prevention. Currently, Clinicians can diagnose TB in several ways, including sputum smear microscopy, immunological methods, rapid molecular tests, culture-based techniques, as well as with the use of chest X-rays, CT scans, polymerase chain reaction (PCR) and real-time PCR. Although these are the versatile approaches designed and developed by the researchers for rapid and accurate diagnosis but still there is need to design rapid, highly sensitive, and cost-effective TB test [7–9].

In this scenario nanomaterials are found to be gifted substitute to antibiotics and diagnostic tools. Several studies revealed the effective of nanomaterials for TB [10, 11]. Previously, silver nanoparticles (SNPs) have been shown excellent antimicrobial results against TB, interacting with DNA of bacteria and disturbing the metabolic processes of bacteria [12]. Besides this, some researchers demonstrated that antibacterial effects of SNPs are due progressive oxidation of the silver (Ag) ions. Similarly, other nanomaterials like silica nanoparticles have been also shown promising effects in this context [13, 14].

Gold nanoparticles (GNPs) are promising nanomaterials to diagnose and treat TB, previously it was explained that GNPs can be efficiently employed for diagnostic purposes due to their calorimetric analysis. The GNPs colloidal aggregation can achieve colorimetric sensing of TB due to surface plasmon resonance [6, 14, 15]. The reduction in the distance of adjuvant GNPs results in the red shifting of NP’s surface plasmon resonance red shifts, subsequently, it causes the colour change of the sample from wine red to blue. The alteration in the colour of the GNPs colloidal solution for absorption-based colorimetric detection of a target sample that directly or indirectly triggers GNPs aggregation or re-dispersion provides practical diagnostic platform [16, 17]. Previously, a nucleic acid biosensor that utilized the GNPs adapted with polynucleotides was established, there were target-molecule concentration dependant alterations in the NPs solution upon addition of target-molecule samples, featuring complementary base-pairing. These colour-changes were visible and could be observed by naked eyes, or could be observed using a UV − vis spectrophotometer [18–20].

Due to unique surface characteristics and biocompatibility, mesoporous silica nanoparticles (MSNs) have fascinated high curiosity to design nano-drug delivery systems [21]. The highly ordered network and porous surface make them ideal candidate for nanomedines. For biomedical applications MSNs can be pooled with -organic and -inorganic substances. MSNs can also be used as a carrier for antimicrobial agents. The major pathology of the TB is kind of pulmonary infection, where MSNs have been proved efficient therapeutic cargos for aerosol delivery and are promising choice for treating respiratory diseases [22–25].

In the current study, we have fabricated MSNs that contains GNPs, to establish a rapid diagnostic as well as antimicrobial approach. The GNPs can enhance the antimicrobial effects of the MSNs and would provide synergistic anti-mycotic activity. To the best of our knowledge, we for the first time are reporting MSNs containing GNPs for treatment and diagnosis of TB. This approach could be a promising cost-effective clinical slant for TB diagnosis and treatment.

## Materials and methods

### Materials

The chemicals e.g. methyl alcohol, 2-propyl alcohol, ethyl alcohol, Sodium borohydride, resazurin sodium salt powder (resazurin) and tri-sodium citrate, were supplied by Sigma-Aldrich (St. Louis, MO) unless otherwise stated. Tetraethyl-orthosilicate (TEOS), cetyl-trimethyl-ammonium bromide (CTAB), Hydrogen tetrachloroaurate (III) trihydrate (HAuCl4·3H2O, 99%) was obtained from Acros Organics (Geel, Belgium). N-(amino-ethyl)-amino-propyl trimethoxysilane (TSD) was supplied by ABCR GmbH y Co.KG. All other chemicals were of the uppermost quality and used as received. Milli-Q water was used in all experiments.

### Synthesis of MSNs@GNPs

In the experimental, GNPs were first synthesized with average particles size of approximately 14 nm. Generally, in 500 mL of D/water 0.1 g of HAuCl4·3H2O was added and dissolution was carried out upon heating and boiling under stirrer. Sodium citrate 1%, solution was subsequently added and further stirred for half an hour. The GNPs were collected by centrifugation, and constantly stirred with 10 mL of PEO-SH overnight to allow ligand exchange. The modified-GNPs were collected by means of centrifugation and re-dispersed in ethanol (1 mg/mL) [13]..

MSNs framework was synthesized followed by incorporation of GNPs. In a typical process, 300 mg of CTAB was heated to 75 °C and stirred constantly in mixture of 110 mL D/water and 2 M NaOH. Subsequently, 8 mL of TEOS solution in ethanol was added to CTAB solution dropwise along with the GNPs in ethanol. The resultant mixture was constantly stirred for 15 min form mesoporous silica containing gold MSNs@GNPs. Calcination was then carried out to remove surfactant and obtain mesoporous silica containing gold MSNs@GNPs. MSNs@GNPs were obtained through centrifugation and repeatedly washed with water and ethanol. In final step, the MSNs@GNPs solid fraction was subjected to thermal treatment where NPs were heated up to 600 °C for 3 h and constant rate of air stream was maintained [17].

### Characterization of MSNs@GNPs

The MSNs@GNPs were characterized for their surface morphology and particle size through transmission electron microscopy (TEM) using a transmission electron microscope (TEM; HT7700, Hitachi, Japan). Hydrodynamic size of the GNPs was calculated by dynamic light scattering (DLS) technique using Malvern zeta sizer. The ultraviolet–visible (UV–Vis) spectrum absorption of GNPs and MSNs@GNPs were recorded by UV–Vis spectrometer (Analytik Jena, Germany).

### Mycobacteria culture conditions

*M. tuberculosis H37Rv strain (Mtb)* were supplied by ATCC. Middlebrook 7H9 growth media supplemented with 0.05% Tyloxapol, glycerol 0.5%, catalase, 1% oleic acid, and OADC supplements was used for the culturing of *Mtb*. *Mtb* culturing was performed at 37 °C incubation in T75 culturing bottles. Macrophages (J774 A.1 cell line) were supplied by ATCC and growth cultures were maintained in Dulbecco’s modified eagle medium (DMEM) supplemented with 10% FBS and 1% streptomycin at 37 °C and 5% CO_2_.

### Minimal inhibitory concentration assay

The antimicrobial efficiency of the MSNs@GNPs was evaluated by minimal inhibitory concentration assay (MIC), performed in 96-well plates. MIC values are noticed as the minimum concentration which required to inhibit the growth of bacteria. *Mtb* was grown at influence of 1.5 х 10^4^ bacteria per mL and specified concentrations of MSNs, MSNs@GNPs were added. The plates were then incubated for 20 days at 37 °C. The growth of *Mtb* was noticed at 5, 10, 15, and 20 days via measurement of OD value at 570 nm. Resazurin method was employed in order to determine the MIC values of the nanoparticles. The *Mtb* with nanoparticles were incubated for the mentioned time duration and then 100 μL solution of 0.01% Wt/Vol was added to every well and incubated for 24 h at 37 °C. The viability of the bacteria was noticed by the change of colour from dark blue (oxidized) to pink colour (reduced) [25].

### Cytotoxicity assay

The cytotoxicity of GNPs and MSNs@GNPs in murine macrophages was evaluated by using MTT assay. Macrophages were grown (J774 A.1 cell line) at 37 °C, when morphology became normal GNPs and MSNs@GNPs were added in concentrations (0, 1, 5, 10, 50, and 100 μg/mL). For the positive control group, complete DMEM containing 0.64% phenol was added. The cells were incubated for 24 h with GNPs and MSNs@GNPs and in last 4 h 20 μL MTT solution (5 mg/mL in PBS) was added to every well. Then media was removed and 150 μL DMSO was added to each well and shaken for 15 min to dissolve formazan ring. The absorbance was read at 570 nm by microplate reader.

$$\%\;Cell\;Viability=\frac{Sample-Blank}{\left(Control-Blank\right)}\mathrm x\;100$$

### MSNs@GNPs colorimetric assay conditions

The colorimetric assay was performed to analyse the detection of *Mtb* with MSNs@GNPs. The MSNs, GNPs, and MSNs@GNPs was performed in a total of 40 μL (2.5 nM) volume in PBS (phosphate buffered saline) 10 mM at pH 8, with control group (negative DNA of *Mtb*) and *Mtb* positive samples (10 μg/mL Mtb DNA). After 10 min of denaturation at 95 °C, the mixtures were allowed to stand for 30 min at room temperature and MgCl_2_ was added to a final concentration. Afterwards, 15 min later at room temperature for colour development, the mixtures and the blank were assayed by UV–visible spectroscopy in a microplate reader (Tecan Infinite M200) [15].

### Statistical analysis

All the experiments were performed at least in thrice. The data for MIC showed average of at least three experiment. Results were analysed by student t test and one-way ANOVA where statistical analysis was applicable.

## Results and discussion

### Synthesis of materials

**Fig. 1:**
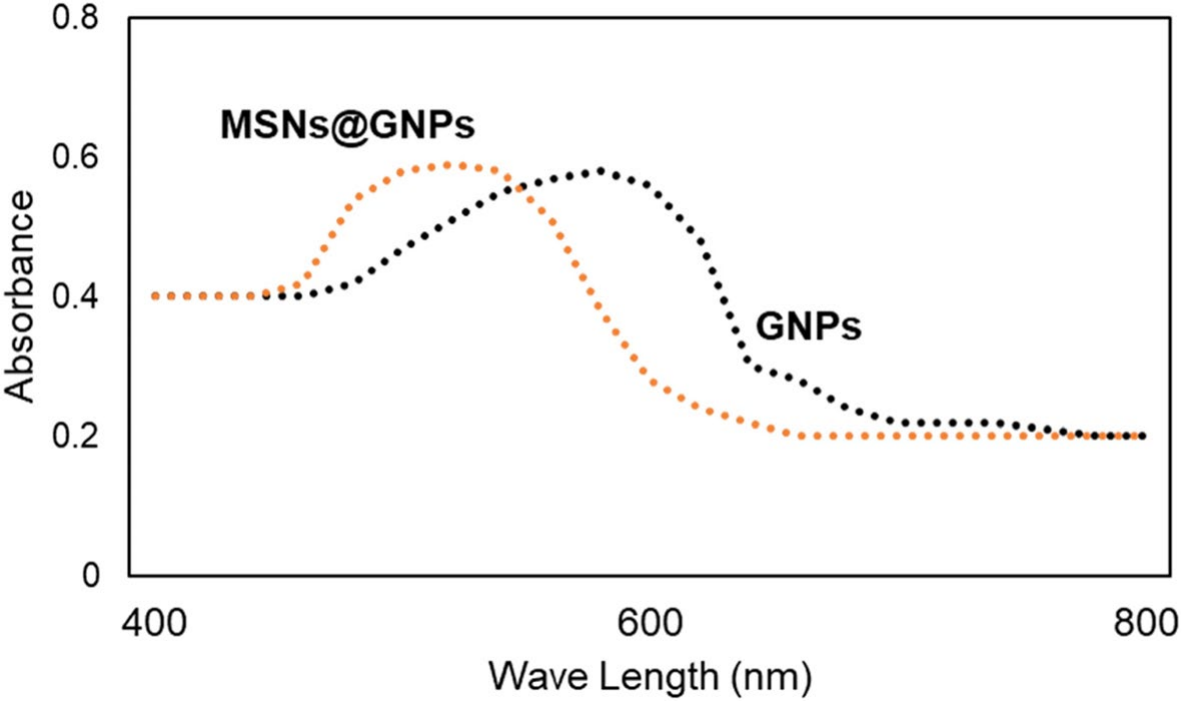
Physical characterization of nanoparticles. UV visible spectrum of the synthesized GNPs and MSNs@GNPs

The GNPs and MSNs@GNPs were successfully synthesised and the verified by UV-visible spectroscopy. The Comparison of UV absorption peaks showed that the maximum absorption wavelength of GNPs was recorded at 580 nm while it was recorded 500 nm for the MSNs@GNPs as shown in Fig. 1. The results are consistent with previously reported data and showed the successful fabrication of the MSNs@GNPs. The reduction in the absorption wavelength is also a key indicator that the MSNs@GNPs are successfully designed and GNPs are entrapped in the pores of the MSNs. This complex can be successfully employed for the delivery and can synergize the antimicrobial effect of both nanomaterials. Besides the synergistic antimicrobial effects, at the same time MSNs@GNPs can be utilized for the TB diagnostic purposes as well, due to the calorimetric properties of the GNPs as reported earlier [6]. So, MSNs@GNPs can be diagnostic and TB rehabilitation approach towards nanotechnology.

### Characterization of MSNs@GNPs

**Fig. 2:**
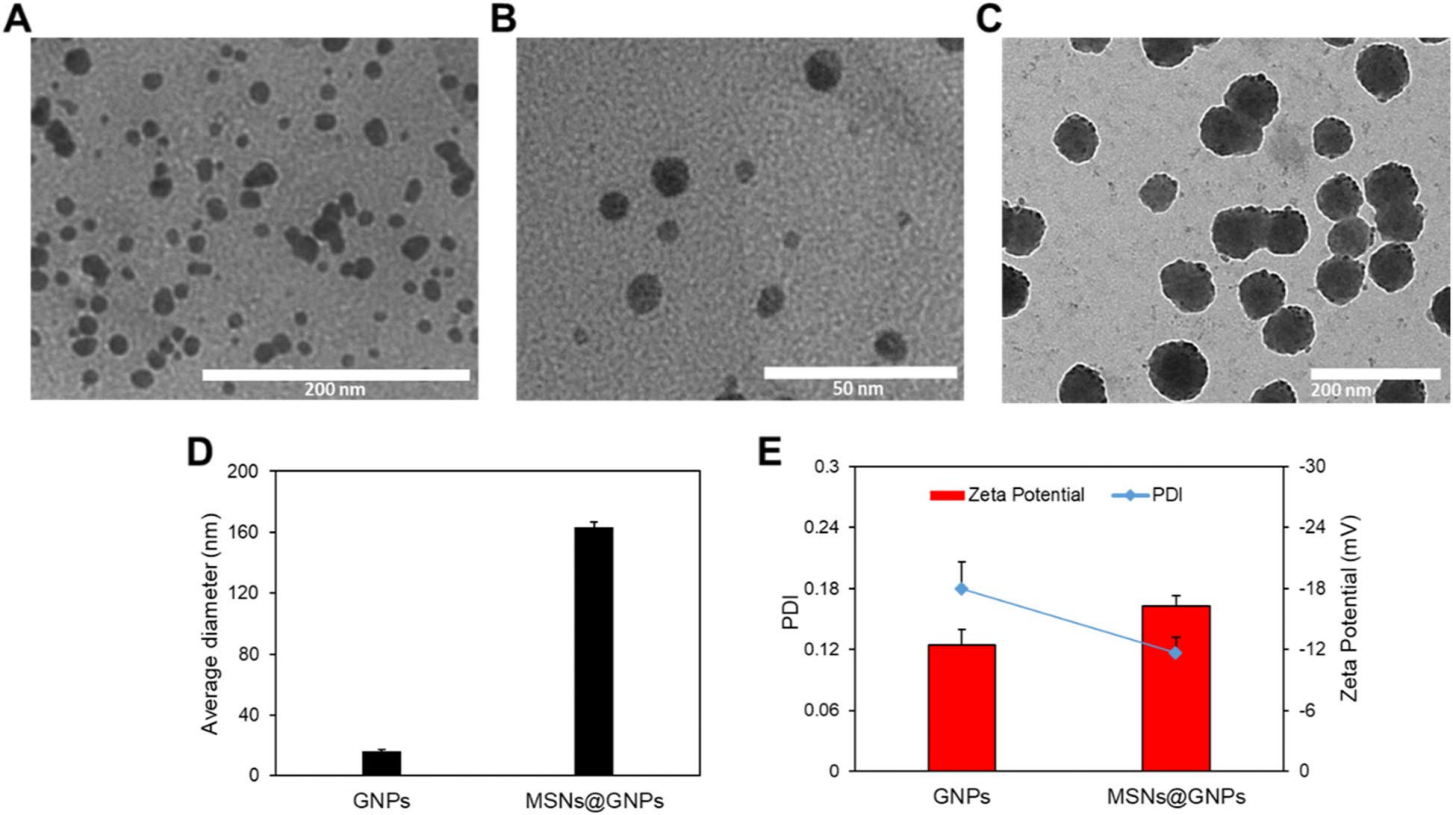
Physicochemical characterization of the nanoparticles. **A**, **B** TEM images of the GNPs, scale bar is 200 and 50 nm respectively. **C** TEM images of the MSNs@GNPs, scale bar is 200 nm. **D** Hydrodynamic size distribution of the GNPs and MSNs@GNPs measured by dynamic light scattering technique. **E** PDI and zeta potential of the GNPs and MSNs@GNPs measure by dynamic light scattering technique. Data are represented as mean ± SD

The MSNs@GNPs surface morphology and size were characterized by the transmission electron microscope (TEM). The TEM images showed round surface morphology for the GNPs with size of approximately 12 ± 0.67 nm as shown in Fig. 2A and B. while the MSNs@GNPs showed comparably porous surface pattern having size of approximately 87 ± 2.3 nm as shown in the Fig. 2C. The porous surface of the MSNs is furrowed with the GNPs and create slightly embedded morphology that is obvious in the Fig. 2C. The hydrodynamic size of the nanoparticles was measured by dynamic light scattering technique, that revealed ≈163 nm size for the MSNs@GNPs while ≈16 nm for GNPs as shown in the Fig. 2D. The slight increase in the diameter might be due to hydrodynamic layer in aqueous phase which is negligible in case of GNPs while prominent in case of the MSNs@GNPs. The PDI and zeta potential were recorded less than 0.2 and negative respectively, for both the nanoparticles as shown in the Fig. 2E. The PDI less than 0.2 is generally considered well enough for the uniform distribution of the nanoparticles, so the DLS results revealed excellent characterization of the nanoparticles [15, 19] and supposed that MSNs@GNPs can be further characterized for in vitro data.

### Minimal inhibitory concentration assay and toxicity assay

**Fig. 3:**
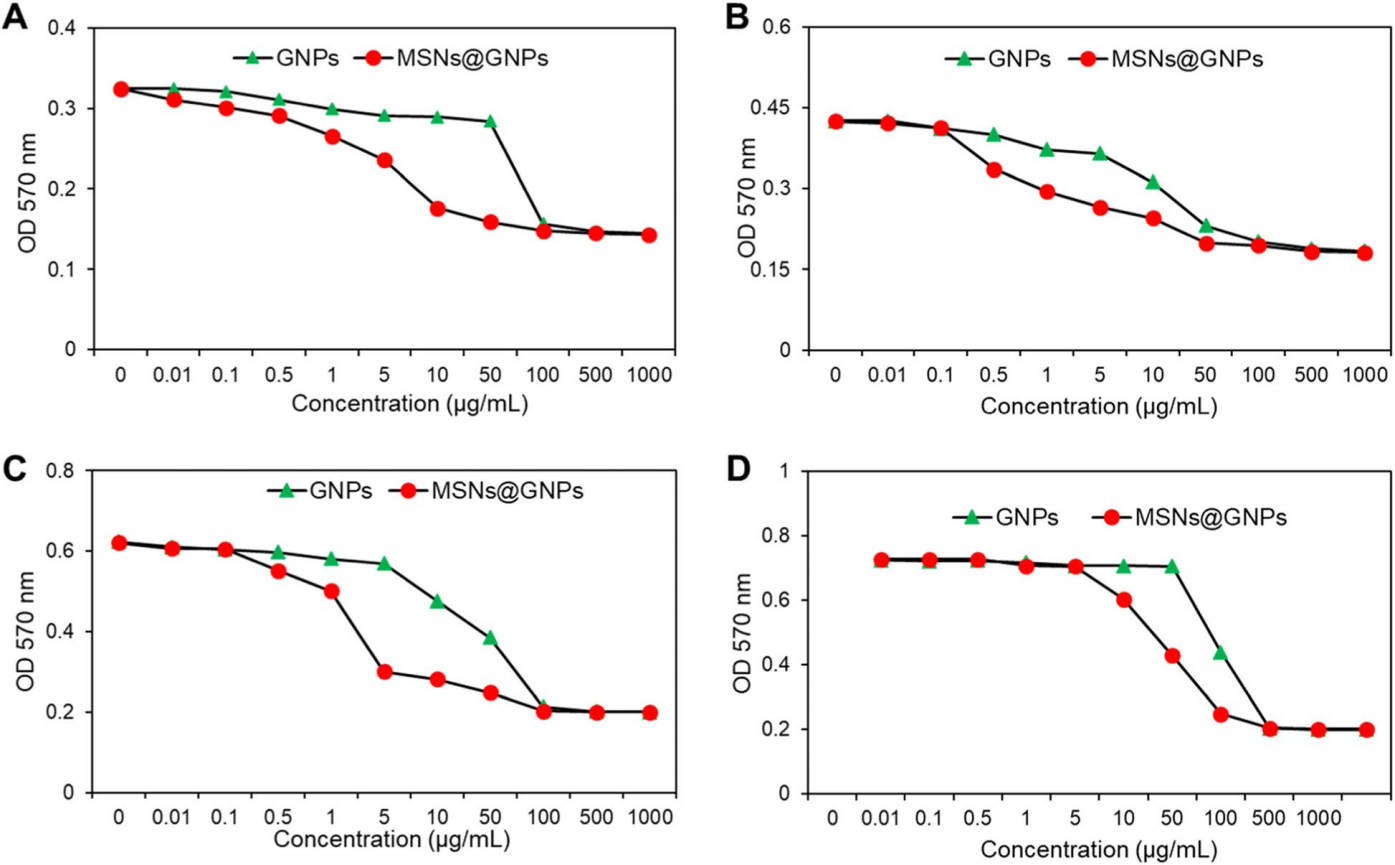
Antimicrobial analysis of MSNs@GNPs. **A** MIC assay of GNPs and MSNs@GNPs after 5 days. **B** MIC assay of GNPs and MSNs@GNPs after 10 days. **C** MIC assay of GNPs and MSNs@GNPs after 15 days. **D** MIC assay of GNPs and MSNs@GNPs after 20 days

To determine the antimicrobial efficiency of the nanoparticles and their complex, minimum inhibitory concentration assay was performed. Antimicrobial efficiency was measured at 5, 10, 15 and 20 days after incubation of GNPs and MSNs@GNPs after incubation with *Mtb* culture. The MIC value recorded for MSNs@GNPs was comparably lower than GNPs at all-time points as shown in Fig. 3. After 20 days the MIC for GNPs was 54 μg/mL and 11 μg/mL for MSNs@GNPs. The significant decrease in the MIC value for MSNs@GNPs showed that the composition can efficiently compromise the integrity of the *Mtb* and can effective approach while treating the TB. GNPs is useful nanosystem for the calorimetric diagnosis of the TB but couldn’t kill the *Mtb* efficiently and can’t be employed for the treatment of TB [14]. On the other hand, MSNs efficiently degrade *Mtb* culture and produce serious toxic effects to *Mtb* cultue. The combination MSNs@GNPs in this case is more efficient and produce synergistic effect to some extent. The results revealed that this nanosystem can be a clinical translation for the patients suffering from TB. The cytotoxic effects of GNPs and MSNs@GNPs were evaluated on macrophages (J744A.1 cells). The results showed very minimal toxicity at all concentrations as shown in Fig. S1, the results that nanoparticles are safe and have no toxicity for macrophages. It might be due to less uptake and engulfment of the nanoparticles by macrophages.

### MSNs@GNPs colorimetric assay conditions

**Fig. 4:**
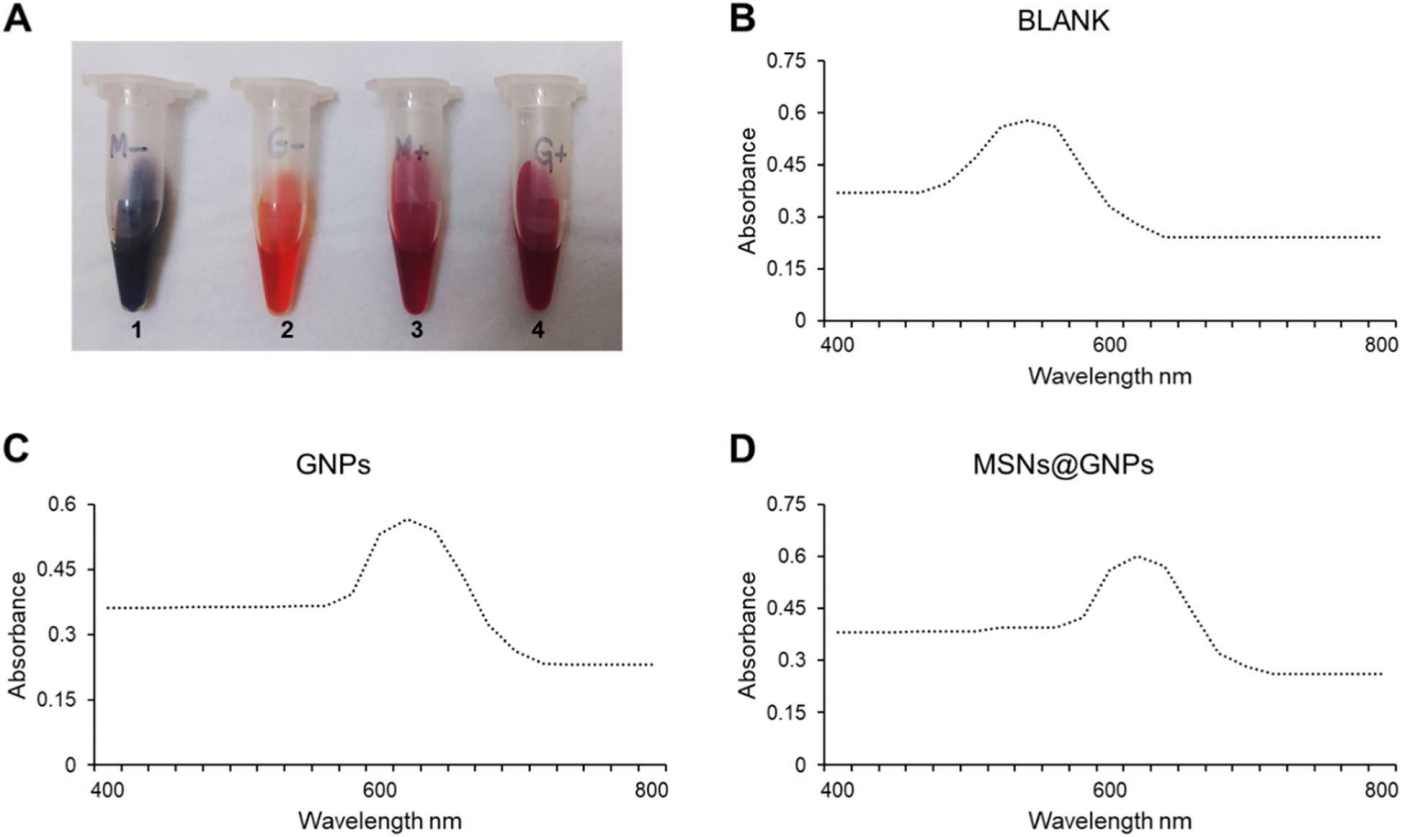
Calorimetric analysis of MSNs@GNPs. **A** Images of visible quenching of *Mtb* with GNPs and MSNs@GNPs where 1 is *Mtb* negative MSNs@GNPs sample, 2 is *Mtb* negative GNPs sample, 3 is *Mtb* positive MSNs@GNPs sample, and 4 is Mtb positive GNPs sample. **B** Absorption spectrum of the *Mtb* cell culture. **C** Absorption spectrum of the GNPs with *Mtb* cell culture. **D** Absorption spectrum of the MSNs@GNPS sample with the *Mtb* cell culture

The diagnostic efficiency of the MSNs@GNPs was evaluated by colorimetric assay as described earlier. There are many tools and techniques for the detection of TB directly and *Mtb* susceptibility. However, these techniques are difficult, very expensive and difficult to use in normal routine clinical practice. So, here we presented a dual approach for TB treatment as well as TB diagnosis by loading GNPs into MSNs. The Fig. 4A showed the quenching of both the *Mtb* positive samples comparably similar while found different for the *Mtb* negative samples. While the absorption spectra in Fig. 4B, C and D revealed that GNPs and MSNs@GNPs *Mtb* positive sample have comparable absorption peaks as compared to the blank *Mtb* samples. These results strongly suggest and recommends the use of MSNs containing GNPs for rapid diagnosis and treatment of the TB. This approach can be further applied clinically. MSNs@GNPs is cost effective and routinely applicable approach in rapid diagnosis and treatment of TB.

In the present work an attempt was made to utilize mesoporous silica nanoparticles and gold nanoparticles for the rapid diagnosis as well as treatment of the tuberculosis. Previously mesoporous silica nanoparticles have been proven to have effective MIC for *mycobacterium tuberculosis* while calorimetric analysis of GNPs shown their effect diagnostic capability. Here in, we combine both the nano systems to develop nano system for rapid diagnosis as well as treatment of tuberculosis. We successfully declared in vitro that mesoporous nanoparticles containing gold can be used for dual purposes of rapid diagnosis as well as treatment of tuberculosis. However, in the near future the designed approach will be further utilized and analysed for clinical samples from tuberculosis patients. The fabricated nano system can be a rational clinical slant for rapid diagnosis and rehabilitation of tuberculosis.

## Supplementary Information

**Additional file 1.**

## Data Availability

Data sets can be available upon reasonable request from corresponding.
