# Supplementary material for: Novel mesoporous silica nanocarriers containing gold; a rapid diagnostic tool for tuberculosis
Source: BMC Complement Med Ther. 2021 Nov 5;21:277. doi: 10.1186/s12906-021-03451-7 (PMC8569953; doi:10.1186/s12906-021-03451-7)
Supplement: Supplementary file 1 — Additional file 1. [file 12906_2021_3451_MOESM1_ESM.docx]

Supporting Material

**Novel Mesoporous Silica Nanocarriers Containing Gold; A rapid diagnostic tool for Tuberculosis**

Sun Chang^#^, Zhang Xiaoying^#^, Wang Jialu, Chen Yahao, and Meng Cunren^*^

Xinjiang Medical University First Affiliated Hospital Urumqi, Xinjiang 830011, China

Corresponding author email (^*^); [mcr_123@163.com](mailto:mcr_123@163.com), mcr_1231@hotmail.com

(^#^) these authors contributed to the work equally.


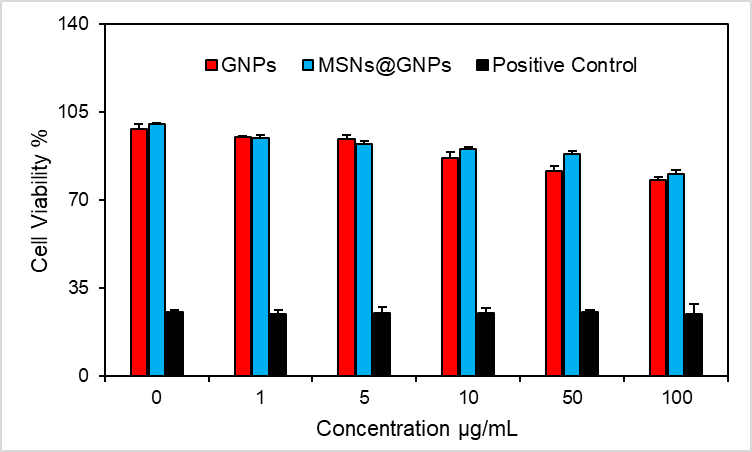


**Supporting Figure 1.** Evaluation of cytotoxicity of GNPs and MSNs@GNPs in Macrophages. Data were presented as Mean ± SD, n=3.
